# Evaluation of pseudoephedrine pharmacy sales before and after mandatory recording requirements in Western Australia: a case study

**DOI:** 10.1186/s13011-016-0075-0

**Published:** 2016-08-30

**Authors:** Hendrika Laetitia Hattingh, Janki Varsani, Leila Ataei Kachouei, Richard Parsons

**Affiliations:** 1 School of Pharmacy, Faculty of Health Sciences, Curtin University, GPO Box U1987, Perth, WA 6845 Australia; 2 Adjunct Senior Research Fellow, School of Pharmacy, Griffith University, Gold Coast, Australia

## Abstract

**Background:**

A community pharmacy real-time electronic recording program, ProjectSTOP, enables Australian community pharmacists to verify pseudoephedrine requests. In Western Australia the program was available for voluntary use from April 2007 and became mandatory November 2010. This case study explores the effectiveness of the program by reviewing the total requests for pseudoephedrine products, and the proportion of requests which were classified as ‘denied sales’ before and after mandatory implementation. Seasonal and annual trends in these measures are also evaluated.

**Methods:**

ProjectSTOP data recordings for Western Australia pharmacies between 1 December 2007 and 28 February 2014 were analysed. Data included a de-identified pharmacy number and date of each pseudoephedrine product request. The total number of requests and sale classification (allowed, denied, safety, or not recorded) were calculated for each month/pharmacy. The potential influence of mandatory reporting using ProjectSTOP was investigated using a Regression Discontinuity Design. Correlations between sales from the same pharmacy were taken into account by classifying the pharmacy number as a random effect. The main effects of year (continuous variable), and season (categorical variable) were also included in the model.

**Results:**

There was a small but steady decline in the total requests for pseudoephedrine per month per 100,000 population (per pharmacy) from the time of mandatory reporting. The number of denied sales showed a steady increase up until mandatory reporting, after which it showed a significant decline over time. Total sales were heavily influenced by season, as expected (highest in winter, least in summer). The seasonal pattern was less pronounced for denied sales, which were highest in winter and similar across other seasons. The pattern over time for safety sales was similar to that for denied sales, with a clear change occurring around the time of mandatory reporting.

**Conclusion:**

Results indicate a decrease in pseudoephedrine product requests in Western Australia community pharmacies. Findings suggest ProjectSTOP has been successful in addressing suspicious sales and potential diversion however ongoing data review is recommended.

## Background

Pseudoephedrine is an effective nasal decongestant included in many cough and cold products that are available over-the-counter (OTC) in Australian community pharmacies [1, 2]. It is similarly available without a prescription in many overseas countries [3, 4]. However, pseudoephedrine can be diverted (utilised as a precursor in methamphetamine production in clandestine laboratories) illegally into methamphetamine [5, 6], which is a more potent form of the drug amphetamine and the most common type of illicit amphetamine available in Australia [7]. The diversion is an ongoing concern [8, 9] because methamphetamine, which can be inhaled, smoked or injected, is a highly addictive substance [10]. In 2006, the Australian Federal Police reported an upsurge in the diversion of pseudoephedrine products in clandestine laboratories [11]. The most recent Illicit Drug Data Report showed that the number and volume of amphetamine detections at Australian borders were the highest on record during 2012–2013 [12] and amphetamine use is therefore a growing problem.

Various research projects and government reports have highlighted that the diversion of pseudoephedrine is an alarming public health concern [13–15]. Of additional concern is the fact that illicit methamphetamine manufacturing can have detrimental effects on the individuals involved in the manufacturing operations as well as innocent bystanders and individuals residing nearby [8]. A review of the literature by White et al. highlighted the injuries associated with the pseudoephedrine-to-methamphetamine diversion process with some reported injuries being severe, ranging from pulmonary effects to chemical burns [16]. Caldicott and colleagues also reported the harms associated with the chemicals used in the diversion process which are explosive in nature and have been implicated in fatal accidents [9]. They reported that some accidents have resulted in environmental damage that impacted on local agriculture and required costly site clean-ups. Also, illegal diversion activity has been documented to increase hospitalisations and lead to time and money spent in legal prosecutions of those involved in these activities [5].

In an attempt to prevent the diversion of pseudoephedrine to methamphetamine various regulatory restrictions have been introduced in Australia throughout the last decade. These regulatory restrictions are specifically aimed at individuals referred to as ‘pseudo-runners’ who visit pharmacies to purchase pseudoephedrine products that are then used to manufacture methamphetamine [17]. The regulatory restrictions involved changes in federal as well as jurisdictional legislation. The combination of federal and jurisdictional regulatory restrictions was needed as reports from law enforcement agencies in 2004 indicated that the majority of illicit methamphetamine available at that stage was produced from pseudoephedrine products obtained from community pharmacies [18]. It was therefore necessary to implement national scheduling changes that addressed OTC pack sizes and the need for a pharmacist’s involvement in supplies as well as additional consumer identification and recording requirements, implemented by jurisdictions.

At federal level, the Therapeutic Goods Administration rescheduled OTC products containing pseudoephedrine in 2006 from Schedule 2 (Pharmacy Medicine) to Schedule 3 (Pharmacist Only Medicine), thus classifying them as non-prescription items that can only be supplied with direct involvement of a pharmacist [19]. This change included prohibition of advertising pseudoephedrine products to the general public in a further attempt to reduce inappropriate use [1]. Additionally, all liquid preparations that contained over 800 mg and packages of products that contained over 720 mg of pseudoephedrine were rescheduled to Schedule 4 (Prescription Medicine). These changes were made to reduce pseudoephedrine availability to the public and formalise pharmacists’ role in determining whether a consumer has a therapeutic need for a pseudoephedrine product. Similar changes have been introduced internationally [20, 21] with varied results [22].

At jurisdictional level all Australian states and territories made changes to drugs and poisons legislation to address the management of requests, selling and recording of pseudoephedrine. Changes to the Poisons Regulations 1965 (WA) in July 2007 incorporated the requirement to verify the purchaser’s identification and record the sale of all pseudoephedrine products [23]. All other jurisdictions except Tasmania and Victoria introduced similar legislation [24]. Queensland also incorporated an additional approach to combat inappropriate sales through the introduction of a linked electronic medication recording system called ProjectSTOP in November 2005 [25]. ProjectSTOP is a web-based system that allows the pharmacist to enter details of a potential customer from approved standard identifying documents (i.e. drivers licence). If a set system threshold to access pseudoephedrine products is breached the system displays previous recent sales, therefore allowing the pharmacist to review previous sales of pseudoephedrine-containing products, as well as instances where sales may have been denied, for this person. As it is continually updated in real time, ProjectSTOP assists pharmacists in the decision to supply a pseudoephedrine product or not, regardless of whether it has been prescribed or not [26]. The system was developed by GuildLink (a wholly owned subsidiary company of the Pharmacy Guild of Australia) and the Chemical Diversion Desk of the Queensland Police Service [27], with the express purpose of preventing pseudo-runners from accessing pseudoephedrine.

Each recording in the linked electronic medication recording system includes the pharmacy identification (ID) number, postcode, state, date of transaction, product formulation, quantity and ingredients as well as customer details. Customer details include date of birth, full name, full address and photo ID type i.e. driver’s license or proof of age card. A check of the customer ID is then conducted through ProjectSTOP which results in either a *match* or *no match*. A *no match* result indicates that the customer’s ID has not been entered into the system within the threshold period whereas a *match* indicates a recent transaction that breached the threshold period or a recent pseudoephedrine product purchase. Depending on the customer’s record, the sale of a pseudoephedrine product could then be *allowed*, *denied* or classified as a *safety sale* (where the pharmacy staff may supply the product out of safety concerns). The program can also allocate to a category called *not recorded* to be used when pharmacy staff did not record any customer details but still supplied the product. There is evidence that the real time recording system enables recognition of suspicious and legitimate requests of pseudoephedrine-containing products [28, 29], which may be used by the police and health agencies to track non-legitimate pseudo-runner users.

ProjectSTOP was introduced across other Australian states during 2007 as a voluntary reporting tool. Real time recording subsequently became mandatory in Queensland, South Australia, Western Australia (November 2010) and the Australian Capital and Northern Territories. Victoria, Tasmania and New South Wales still operate under a voluntary reporting system [24]. A recent comparison of pharmacy-reported pseudoephedrine sales between Queensland and Victoria showed an increased uptake and use of the recording system in a jurisdiction with mandatory real-time recording requirements compared to a jurisdiction with voluntary recording requirements [29].

The purpose of the present study was to investigate whether the introduction of mandatory real-time recording in Western Australia (WA) influenced the number of requests for pseudoephedrine (per 100,000 population) from community pharmacies, or the proportion of requests which were *denied*. Seasonal differences and trends over time were taken into account.

## Methods

Data entered into ProjectSTOP by WA community pharmacies were provided by GuildLink following a confidentiality agreement between Curtin University and GuildLink. This study was conducted between July and October 2014. Low-risk ethical approval was granted by Curtin University (PH-14-14).

### Data preparation

More than 2.5 million retrospective pharmacy records were received from GuildLink covering WA community pharmacy sales between 1 December 2007 and 28 Feb 2014. The population estimates for WA were obtained from the Australian Bureau of Statistics [30]. Monthly estimates were obtained by interpolating between census dates.

The total number of requests for pseudoephedrine and the proportion of those requests which were denied were calculated for each month. The total requests per 100,000 population was calculated by reference to the monthly population figures. The seasons (southern hemisphere) were defined according to the month, so that December, January, February were classified as Summer; March, April, May as Autumn; June, July, August as Winter; and September, October, November as Spring. Sales of pseudoephedrine were expected to follow a seasonal pattern with most sales occurring during winter months.

### Statistical methods

Total monthly sales and denied sales per pharmacy were calculated and summarised in graphs after adjusting for total population figures (sales expressed as number per 100,000 population). The potential influence of mandatory reporting using ProjectSTOP was investigated using a Regression Discontinuity Design [31]. In particular, a Generalised Estimating Equation was used with the monthly number of sales at each pharmacy as dependent variable and timing relative to the date of mandatory reporting (pre- or post-) was included as an independent variable. Correlations between sales from the same pharmacy were taken into account by classifying the pharmacy number as a random effect. This model is very similar to the Poisson regression model, but the standard errors are corrected for the within-pharmacy correlations. The main effects of year (continuous variable), and season (categorical variable) were also included in the model, which was of the form:

$$ \mathrm{Sales} = \upalpha + {\upbeta}_1\ *\ \mathrm{Season} + {\upbeta}_2\ *\ \mathrm{Year} + {\upbeta}_3\ *\ \mathrm{Mandatory} + \upgamma\ *\ \mathrm{Year}\ *\ \mathrm{Mandatory} + \upepsilon $$

In this equation, Sales represents the number of sales in a month at each pharmacy, Season is a categorical variable (summer/autumn/winter/autumn), Year is the number of months from the date of mandatory reporting (negative if prior to the date, positive otherwise) divided by 12, and Mandatory is a binary indicator variable which is zero prior to reporting and ‘1’ otherwise. The coefficients (Greek letters) are estimated from the modelling procedure, which also estimates the statistical significance of their contributions to the model fit. In particular, β_3_ indicates if there is any sudden change at the date of mandatory reporting, and γ indicates whether the annual rate of sales appears to change from pre- to post-mandatory reporting. One model was developed with the total number of requests for pseudoephedrine at each pharmacy each month as dependent variable, and a second model used as dependent variable the number of requests which were classified as denied sales. The intercept term in the model (α) included an offset variable defined as the logarithm of the population estimate for WA for the month, so that the rate ratios obtained from the model were adjusted for population growth over the period under study. Results of the regression models were expressed as rate ratios, their 95 % confidence intervals and p-values. Analyses were conducted using the Genmod procedure in the SAS version 9.2 statistical software (SAS Institute Inc, Cary, NC, USA, 2008), and, following convention, a *p*-value < 0.05 was taken to indicate a statistically significant association in all tests.

## Results

**Fig. 1:**
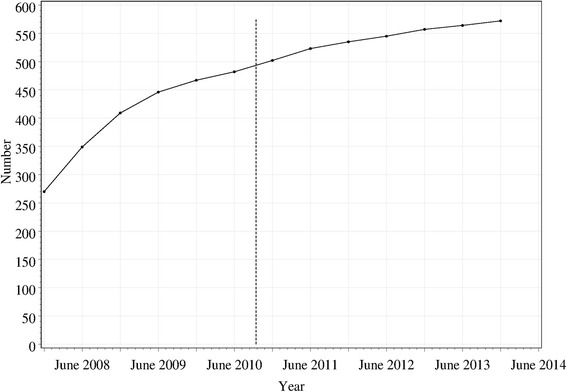
Number of pharmacies reporting data to GuildLink over the period under study. The *vertical dashed line* shows the time at which mandatory reporting was introduced

There were 2,573,905 requests for pseudoephedrine products recorded in the system, from 572 WA community pharmacies between December 2007 and February 2014. The number of pharmacies in the system correlates with the number of community pharmacies in WA [32]. The number of pharmacies reporting data to GuildLink grew from 270 at the start of the period under study, in a moderately linear fashion (Fig. 1). This suggests that the reporting system had been gaining support steadily, prior to it becoming mandatory. Although information is not available on the total number of pharmacies in WA between in 2008–2010, data from the Pharmacy Registration Board of WA that was established in October 2010 indicates that there were 544 pharmacies in June 2011, therefore 523/544 which was a 96.1 % uptake of ProjectSTOP. This increased to 545/563 (96.8 %) and 564/576 (97.9 %) uptake in 2012 and 2013 respectively [33].

Of all requests for pseudoephedrine, 95.7 % were ‘allowed’, 2.2 % were classified as ‘safety sale’, 1.1 % were ‘not recorded’ and 0.9 % were ‘denied’. During the same period, the population of WA increased from 2.14 million to 2.57 million. The crude (unadjusted) average monthly number of requests for products changed from 3.8 per 100,000 population prior to mandatory reporting, to 3.1 per 100,000 population following its introduction.

### Analysis of total requests for pseudoephedrine products

**Fig. 2:**
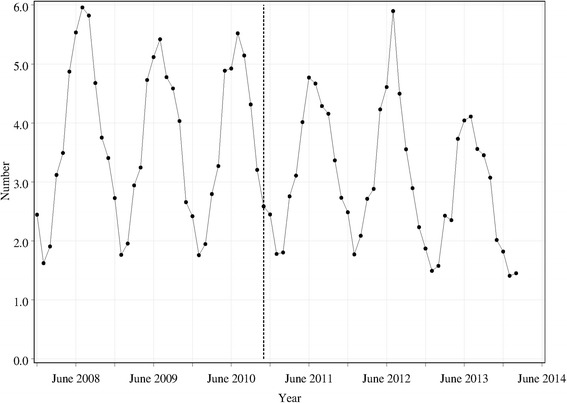
Total requests for pseudoephedrine per pharmacy per 100,000 population per month. The *vertical dashed line* shows the time at which mandatory reporting was introduced

Figure 2 shows the total monthly requests for pseudoephedrine per 100,000 population over the time of the study. There is a very clear seasonal trend, which is confirmed in the regression analysis (Table 1). The monthly requests were highest in winter, and lowest in summer, with spring and autumn intermediate in number. Rates in spring and autumn were similar, but all other pairwise differences in rates between seasons were found to be significantly different. After adjustment for the very significant difference between seasons, there was no apparent change in requests around the time of the mandatory reporting (*χ*^2^ (1) = 0.9; *p* = 0.36). While there was no significant overall trend across time, there did appear to be a significant change in slope of the line from the time of mandatory reporting (*χ*^2^ (1) = 4.2; *p* = 0.0415). The annual rate of change in total sales per population reduced to 0.93 from November 2010.

**Table 1:** Regression analysis for the total requests per pharmacy per month

Variable	Chi-square (DF)	Rate ratio	95 % CI	*p*-value
Season	4741 (3)			<0.0001
Summer		0.57	0.56–0.59	<0.0001
Autumn		0.99	0.98–1.01	0.4587
Winter		1.44	1.42–1.47	<.0001
Spring		1 (reference)		
Mandatory	0.85 (1)	0.98	0.94–1.02	0.3578
Year	2.1 (1)	0.97	0.94–1.01	0.1463
Mandatory Year^a^	4.2 (1)	0.95	0.91–1.00	0.0415
Year (before)^b^		0.97	0.94–1.01	0.1463
Year (after)^b^		0.93	0.91–0.95	<0.0001

Correlations between observations made at the same pharmacies were taken into account as a random effect

^a^Interaction term between year and Mandatory (indicating pre- or post- reporting)

^b^Separate annual rates pre- and post-mandatory reporting

### Analysis of the trends in denied sales

**Fig. 3:**
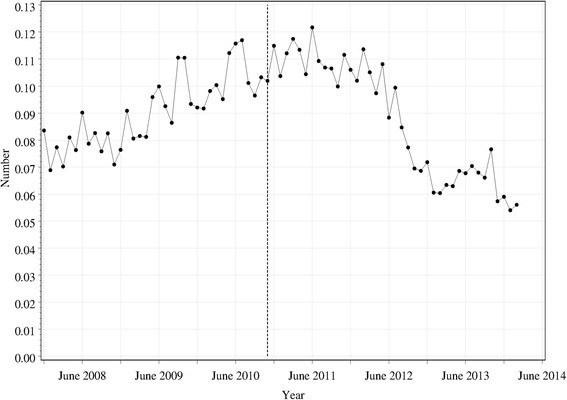
Total number of denied requests for pseudoephedrine per pharmacy per 100,000 population per month. The *vertical dashed line* shows the time at which mandatory reporting was introduced

The number of requests for pseudoephedrine per month which were denied are shown in Fig. 3 (per 100,000 population). The vertical line shows the time at which mandatory reporting was introduced. The regression analysis (Table 2) shows a higher number of denied sales in winter compared other seasons, and pairwise differences show that the rate in winter was significantly higher than that for summer (*χ*^2^ (1) = 8.0; *p* = 0.0048) or spring (*χ*^2^ (1) = 8.0; *p* = 0.0.0047), but not significantly different from autumn (*χ*^2^ (1) = 1.5; *p* = 0.229). There was a significant change in the slope of the line pre- to post-mandatory reporting, with the rate changing from significantly increasing to significantly decreasing. For some 6–8 months after introduction of mandatory reporting, the trend in denied sales was flat, reflected in the results as a discontinuity at the time of mandatory reporting itself.

**Table 2:** Regression analysis for the monthly number of denied sales per pharmacy

Variable	Chi-square (DF)	Rate ratio	95 % CI	*p*-value
Season	12.5 (3)			0.0059
Summer		0.99	0.93–1.05	0.6748
Autumn		1.03	0.96–1.10	0.3674
Winter		1.07	1.02–1.12	0.0047
Spring		1 (reference)		
Mandatory	6.5 (1)	1.17	1.04–1.31	0.0107
Year	14.9 (1)	1.14	1.09–1.20	<0.0001
Mandatory Year^a^	74.3 (1)	0.70	0.64–0.76	<0.0001
Year (before)^b^		1.14	1.09–1.20	<0.0001
Year (after)^b^		0.80	0.76–0.83	<0.0001

^a^Interaction term between year and Mandatory (indicating pre- or post- reporting)

^b^Separate annual rates pre- and post-mandatory reporting

**Fig. 4:**
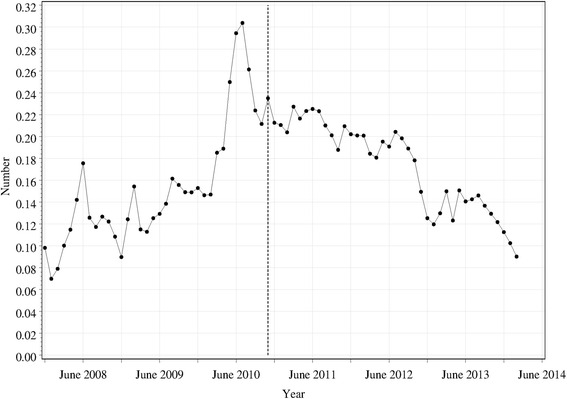
Total number of safety sales of pseudoephedrine per pharmacy per 100,000 population per month. The *vertical dashed line* shows the time at which mandatory reporting was introduced

The pattern for safety sales was similar to total and denied sales (Fig. 4), and the analysis showed a clear change in slope from pre- to post-mandatory reporting (*χ*^2^ (1) = 36.1; *p* < 0.0001). It also showed a significant seasonal affect (*χ*^2^ (3) = 54.6; *p* < 0.0001), similar to that for total sales (rates highest in winter, lowest in summer). There was no discontinuity at the time of mandatory reporting (*χ*^2^ (1) = 0.1; *p* = 0.77), suggesting that there was no significant delay between the increasing trend prior to this date and the subsequent decreasing trend.

## Discussion

There appeared to be a downward trend in the total requests for pseudoephedrine products per month, as well as very significant seasonal differences. The denied sales, on the other hand, showed a significant increase up to the introduction of ProjectSTOP, followed by some 6–8 months of no change and then a significant decrease from that time forward. The downward trend in denied sales started approximately six months after the introduction of ProjectSTOP as a mandatory requirement. Of interest is the plateauing of the trend in denied sales following the mandatory introduction which could suggest that consumers who were previously able to purchase pseudoephedrine-containing products started to have requests denied by pharmacies that did not up to that time participate in ProjectSTOP. This pattern was similar for safety sales. The strong seasonal effect in requests was expected with highest demand for products in the winter and least in summer periods. The seasonal trend in denied sales was much weaker, but similar to total sales with the highest numbers occurring in winter.

A number of factors may have contributed to the decrease in monthly sales per 100,000 population. At a pharmacy level these included the TGA scheduling changes introduced in 2006 and the changes to the WA Poisons Regulations 1965, initially in 2007 with further changes in 2010 to mandate the use of ProjectSTOP from November 2010. These changes increased pharmacists’ and potentially customers’ awareness of the vigilance required in the supply of pseudoephedrine products and the prerequisite to confirm therapeutic need. It prompted pharmacists to be more cautious and attentive in the decision-making process whether to allow or deny pseudoephedrine sales. Of specific relevance is that pseudoephedrine requests and sales information from WA pharmacies were not linked until July 2007 and the use of ProjectSTOP was hence recommended on a voluntary basis to facilitate data sharing. The mandatory requirement to use ProjectSTOP was subsequently introduced in WA in November 2010 which further enhanced WA community pharmacists’ ability to identify suspicious requests for pseudoephedrine-containing products. Additionally, the system also provided better intelligence to police and health agencies regarding pseudo-runners and the identification of rogue pharmacists [26, 34].

A comparison between Queensland, with mandatory requirements to use ProjectSTOP, and Victoria that continues on a voluntary basis, has shown a significant difference in the rate of uptake of the system [29]. The mandatory use of ProjectSTOP should therefore ideally be implemented in all jurisdictions to enable tracking of pseudo-runners that visit pharmacies in different states and territories. The Pharmacy Board of Australia indeed endorsed the use of a real-time online monitoring system, such as ProjectSTOP, in the Board’s *Guidelines on practice*-*specific issues* [35].

Data show that the illegal use of methamphetamine remains an ongoing concern. The 2007 National Drug Strategy Household Survey reported that 6.3 % of Australians aged 14 years and older had used meth/amphetamine, which was relatively high compared to other illicit drugs [36]. The Survey also showed that 2.3 % had used it for non-medical purposes in the previous 12 months. Data from the 2010 National Drug Strategy Household Survey showed a slight decrease in meth/amphetamine use with 2.1 % of Australians aged 14 years and older having used it for non-medical purposes in the previous 12 months [37]. Of specific relevance is that a later report, the 2013 National Drug Strategy Household Survey, showed that about 1.3 million Australians aged 14 years and older (7 %) had used meth/amphetamines in their lifetime and 400,000 (2.1 %) had done so in the last 12 months. This report also showed a rise in the proportion of people using it daily or weekly [38] and hence it is an ongoing problem.

Although the overall use of illicit methamphetamines remained relatively stable there have been statistically significant changes in the type, namely crystal or ice versus powder, and frequency of use [38]. The increased use of crystal methamphetamine is concerning as it is not only more potent and hence can cause more fatalities, it also has increased potential for dependence and chronic physical and mental problems [39]. It seems that there have been an increase in illegal methamphetamine imports since 2010, as reported by the Australian Crime Commission (ACC) [40]. The ACC also reported that this growth has occurred without a concurrent fall in domestic production and clandestine laboratory detections and has been able to identify specific individuals involved in pseudoephedrine sales [40]. However the sales through WA community pharmacies have shown a steady decline. The downward trend in pseudoephedrine sales through WA community pharmacies therefore contributes to the multi-strategy approach to reduce inappropriate supplies through community pharmacies.

An increased awareness of ProjectSTOP in the community may have alerted illicit users to the possibility of being tracked and apprehended by the authorities. This could have dampened intentions of purchasing pseudoephedrine-containing products from community pharmacies, and hence the decrease in the number of denied sales. There is also a possibility that there was a heavier reliance on other cough and cold products such as phenylephrine-based products to treat symptoms. Phenylephrine replaced pseudoephedrine in various products as a means of controlling illicit methamphetamine activity although it has been documented that this may not solve the problem associated with methamphetamine abuse [41] and ongoing vigilance is required. It is also worth mentioning that studies have shown phenylephrine to be less effective than pseudoephedrine as a nasal decongestant and that the barriers imposed on obtaining pseudoephedrine may impact on the quality of life of consumers who have a legitimate need for pseudoephedrine [41, 42]. This adds to the complexity of the regulation of pseudoephedrine sales through community pharmacies.

This evaluation of pseudoephedrine recordings provides insight into the value of the real-time recording software as a means of controlling sales of pseudoephedrine through community pharmacies. The findings from this study may be of interest to legislative authorities and policy makers in terms of the ongoing battle against the illicit manufacture of methamphetamine from pseudoephedrine. The combination of regulatory restrictions at federal and jurisdictional levels could also be useful in the development of controls for other medicines with potential for abuse, such as codeine containing OTC and prescription products.

The study was limited by the scheduling changes that occurred during the time period analysed. These scheduling changes could have influenced pseudoephedrine demand patterns. Another major limitation is that the recording of the sale type is based on the decision of the pharmacist or pharmacist assistant. This decision could be influenced by environmental factors, time constraints, level of experience and personal judgement.

## Conclusion

This study has provided insight into annual requests for pseudoephedrine sales and denied and safety sale trends before and after the mandatory implementation of ProjectSTOP in WA. Seasonal trends show that a higher percentage of sales are denied in the summer, therefore pharmacists are denying sales when there do not seem to be a therapeutic need. The use of ProjectSTOP has been a positive step in identifying suspicious sales from community pharmacies and subsequently combatting the diversion of pseudoephedrine to methamphetamine. However, future studies are required to better assess the impact of the program in reducing illicit activity.

## Abbreviations

ACC: Australian Crime Commission
ID: Identification
OTC: Over-the-counter
WA: Western Australia
